# Retraction: KSHV vFLIP Is Essential for the Survival of Infected Lymphoma Cells

**DOI:** 10.1084/jem.2003146702062021r

**Published:** 2021-02-12

**Authors:** Ilaria Guasparri, Shannon A. Keller, Ethel Cesarman

Vol. 199, No. 7 | 10.1084/jem.20031467 | April 5, 2004

The corresponding author, Dr. Ethel Cesarman, is retracting this article due to multiple instances of splicing and the image manipulation to remove background gel artifacts by duplicating segments of the immunoblots in row six (BCL2 antibody) in Fig. 6. Dr. Cesarman’s research institution, Weill Cornell Medicine (WCM), assembled an investigative committee, which concluded that Fig. 6 represents “falsification” as defined in the WCM Research Integrity Policy because these duplicated images are an example of “changing or omitting data or results such that the research is not accurately represented in the research record.” The multiple instances of splicing and the data manipulation in Fig. 6 were confirmed by image experts at Rockefeller University Press. This retraction supersedes the previous correction of Fig. 4 A of the same article (https://doi.org/10.1084/jem.20031467042606c).

The authors take full responsibility for their mistakes and apologize for their oversight and for any confusion that may have been caused.

